# Doxycycline reverses epithelial-to-mesenchymal transition and suppresses the proliferation and metastasis of lung cancer cells

**DOI:** 10.18632/oncotarget.5842

**Published:** 2015-10-14

**Authors:** Yuan Qin, Qiang Zhang, Shan Lee, Wei-long Zhong, Yan-rong Liu, Hui-juan Liu, Dong Zhao, Shuang Chen, Ting Xiao, Jing Meng, Xue-shuang Jing, Jing Wang, Bo Sun, Ting-ting Dai, Cheng Yang, Tao Sun, Hong-gang Zhou

**Affiliations:** ^1^ State Key Laboratory of Medicinal Chemical Biology and College of Pharmacy, Nankai University, Tianjin, China; ^2^ Tianjin Key Laboratory of Molecular Drug Research, Tianjin International Joint Academy of Biomedicine, Tianjin, China

**Keywords:** doxycycline, antitumor, EMT, metastasis, lung cancer

## Abstract

The gelatinase inhibitor doxycycline is the prototypical antitumor antibiotic. We investigated the effects of doxycycline on the migration, invasion, and metastasis of human lung cancer cell lines and in a mouse model. We also measured the effect of doxycycline on the transcription of epithelial-mesenchymal transition (EMT) markers, and used immunohistochemistry to determine whether EMT reversal was associated with doxycycline inhibition. Doxycycline dose-dependently inhibited proliferation, migration, and invasion of NCI-H446 human small cell lung cancer cells. It also suppressed tumor growth from NCI-H446 and A549 lung cancer cell xenografts without altering body weight, inhibited Lewis lung carcinoma cell migration, and prolonged survival. The activities of the transcription factors Twist1/2, SNAI1/2, AP1, NF-κB, and Stat3 were suppressed by doxycycline, which reversed EMT and inhibited signal transduction, thereby suppressing tumor growth and metastasis. Our data demonstrate functional targeting of transcription factors by doxycycline to reverse EMT and suppress tumor proliferation and metastasis. Thus, doxycycline selectively targets malignant tumors and reduces its metastatic potential with less cytotoxicity in lung cancer patients.

## INTRODUCTION

Antibiotics that target mitochondria have great potential as anticancer drugs. [1]. For example, tetracyclines are cytotoxic to some tumor cells [2] and have been shown to induce apoptosis in osteosarcoma, prostatic cancer cells, and lymphocytes [4, 5]. Doxycycline, a semi-synthetic tetracycline, inhibits matrix metalloproteinase (MMP) activation and cell proliferation [3] and can also interfere with tumor-related protein synthesis in mammalian cells [6–8]. The selective permeability of different mammalian cells to doxycycline led to the hypothesis that it could be used to arrest cell proliferation and treat malignancies. Thus, doxycycline has been used in combination with targeted drugs in clinical trials with patients with advanced cancer.

Lung cancer is the most common cause of cancer mortality worldwide [9]. Tumor recurrence and metastasis are common in lung cancer despite various lines of standard therapy and the introduction of targeted agents. The vast majority of patients with lung cancer fail to respond to tyrosine-kinase inhibitors against the epidermal growth factor receptor (EGFR) [10], and no effective drug is available for small cell lung cancer [11, 12]. Therefore, the development of alternative treatments is urgently needed to improve outcome.

To investigate the therapeutic efficacy and the mechanism of action of doxycycline in lung cancer, we investigated the effects of doxycycline on migration, invasion, and metastasis of various lung cancer cell lines. We also examined its functional interference in EMT. In addition, we used a tumor-bearing mouse xenograft model to investigate doxycycline inhibition of tumor growth *in vivo*.

## RESULTS

### Doxycycline reduces cell viability and alters cell cycle dynamics in human lung cancer cell lines

**Figure 1:**
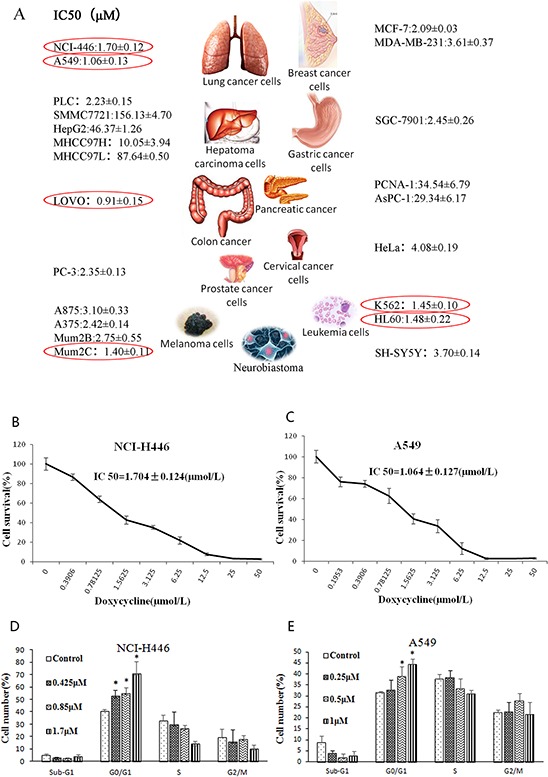
Effect of doxycycline on the cell viability and cell cycle **A.** IC_50_ (μM) dose of doxycycline for different cancer cell lines. Lung cancer cells were more sensitive to doxycycline than all the other cell lines (*P* < 0.05). **B.** Cell survival of NCI-H446 cells and **C.** A549 cells treated with the indicated amounts of doxycycline for 48 h; IC50 = 1.7043 ± 0.1241 μM and 1.0638 ± 0.1203 μM, respectively. **D.** NCI-H446 and **E.** A549 cells treated with different doses of doxycycline for 24 h were evaluated by fluorescence-activated cell sorting (FACS) analysis. Doxycycline induced cell cycle arrest at the G0/G1 phase in both cell lines (*P* < 0.05). Each experiment was performed in triplicate. Results show the means of the three experiments, and the error bars represent standard deviation (*P < 0.05).

Using MTT assay, we determined the effect of 48 h treatment with doxycycline on cell viability of various cancer cell lines. As shown in Fig. 1A, lung cancer cells were more sensitive to doxycycline than most of the other cell lines. NCI-H446 and A549 cells showed sensitivity to doxycycline with IC_50_ values of 1.7043 ± 0.1241 and 1.0638 ± 0.1266 μM, respectively. Proliferation of NCI-H446 (Fig. 1B) and A549 cells (Fig. 1C) was inhibited by doxycycline in a dose-dependent manner.

We also investigated whether doxycycline interferes with the cell cycle. NCI-H446 and A549 cells were treated with different doses of doxycycline for 24 h, followed by cell cycle analysis using flow cytometry. Cells treated with doxycycline started to arrest at G0/G1 phase after treatment for 24 h (Fig. 1D & 1E). After treatment, NCI-H446 cells at G0/G1 accounted for approximately 24% of the total cell population, and A549 cells in G0/G1 population accounted for approximately 44%.

### Doxycycline inhibits lung cancer cell invasion and migration *in vitro*

**Figure 2:**
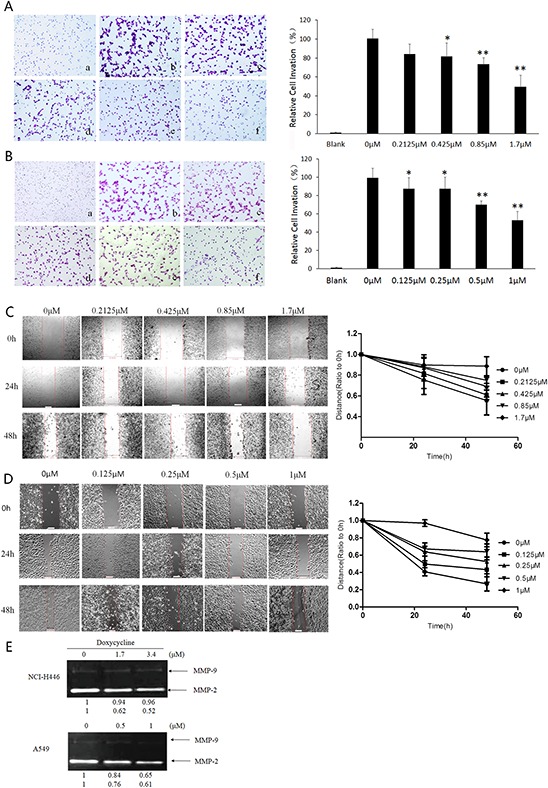
Effect of doxycycline on invasion, migration, and activities of matrix metalloproteinases (MMPs) of NCI-H446 and A549 cells Transwell chambers were used for the invasion assay, and images were taken at 200 × magnification. **A.** NCI-H446 cells were treated with 0 (b), 0.2125 (c), 0.425 (d), 0.85 (e), and 1.7 μM (f) doxycycline for 24 h. No cells were seeded in (a). Doxycycline inhibited invasion of NCI-H446 cells (*P* < 0.05). **B.** A549 cells were treated with 0 (b), 0.125 (c), 0.25 (d), 0.5 (e), and 1 μM (f) of doxycycline for 24 h. No cells were seeded in (a). Doxycycline inhibited invasion of A549 cells (*P* < 0.05). **C.** NCI-H446 cells were incubated in fetal bovine serum (FBS)-free medium containing 0, 0.2125, 0.425, 0.85, or 1.7 μM doxycycline for 24 or 48 h. Doxycycline inhibited the migration of NCI-H446 cells (*P* < 0.05). **D.** A549 cells were incubated in FBS-free medium containing 0, 0.125, 0.25, 0.5, or 1 μM doxycycline for 24 or 48 h. Doxycycline inhibited the migration of A549 cells (*P* < 0.05). **E.** MMP-2 and MMP-9 were downregulated when either cell line was treated with doxycycline. Results are expressed as percentage of control. Similar results were obtained from three independent experiments, each performed in triplicate. Results show the means of the three experiments, and the error bars represent standard deviation (*P < 0.05 and **P < 0.01).

To study whether doxycycline inhibits NCI-H446 and A549 cell invasion we used Matrigel-coated transwell chambers. For both cell lines, when compared with the control group, the number of cell invasions through the Matrigel-coated filter was dose-dependently reduced by doxycycline (Fig. 2A & 2B). Notably, the inhibitory effects of doxycycline on cell invasion were due to its cytotoxic effects, given that cell viability was also decreased at the concentration ranges tested.

Next we assessed the ability of doxycycline to inhibit the migration of NCI-H446 and A549 cells using a wound-healing assay. Confluent cells were scraped with a sterile pipette tip, and the remaining cells were allowed to migrate into the gap created in the absence or presence of doxycycline. Remarkably, after 24 and 48 h treatment, the wound gap of both cell types was wider in the doxycycline-treated groups than in the untreated groups (Fig. 2C & 2D), indicating that doxycycline inhibits motility of both NCI-H446 and A549 cells. The cell growth curves of NCI-H446 and A549 were shown in Fig. S1

The degradation of the extracellular matrix (ECM) and basement membrane are crucial steps in cancer invasion and metastasis and the proteolytic enzymes MMP-2 and MMP-9 are involved in this process. We next measured the secretion of MMP-2 and MMP-9 from NCI-H446 and A549 cells with or without doxycycline treatment. As shown in Fig. 2E, doxycycline inhibited MMP-2 and MMP-9 secretion into the medium in a dose-dependent manner. This finding suggests that doxycycline may reduce lung cancer metastasis by inhibiting the degradation of the ECM and basement membrane.

### Doxycycline inhibits the expression of epithelial markers and changes cellular morphology

**Figure 3:**
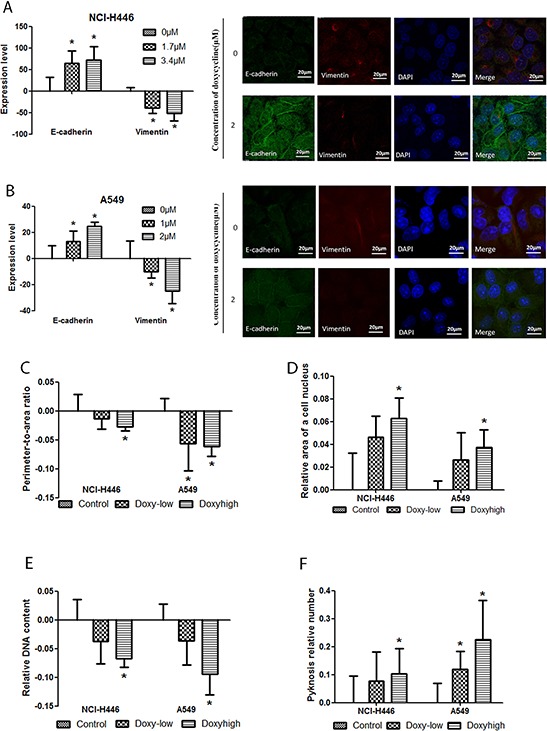
Effect of doxycycline on NCI-H446 and A549 cells visualized with high-content screening (HCS) systems and a fluorescence microscope HCS detection of E-cadherin and vimentin was performed on NCI-H446 and A549 cells treated with 1.7 μM or 3.4 μM and 1 μM or 2 μM doxycycline (respectively) for 24 h. Immunofluorescent staining was done for E-cadherin and vimentin of NCI-H446 and A549 cells treated with 0 or 2 μM doxycycline for 24 h. **A.** In NCI-H446 cells, and **B.** in A549 cells, E-cadherin levels were increased while vimentin levels were decreased with doxycycline treatment. **C.** The ratio of perimeter-to-area was decreased both in NCI-H446 and A549 cells treated with doxycycline. **D.** The relative area of nucleus was increased both in NCI-H446 and A549 cells treated with doxycycline. **E.** The DNA content in a cell was decreased both in NCI-H446 and A549 cells treated with doxycycline. **F.** The number of pyknosis was increased after doxycycline treatment. Standard error bars represent three independent experiments each performed in triplicate (*P < 0.05 compared to the untreated control).

Vimentin and E-cadherin regulate the expression of proteins involved in ECM degradation. Thus, we used immunofluorescent staining to measure the effect of doxycycline on vimentin and E-cadherin levels. NCI-H446 and A549 cells were treated with different doses of doxycycline for 24 h,. In response to doxycycline treatment, vimentin expression decreased, whereas E-cadherin expression increased in both cell lines (Fig. 3A & 3B). We also tested the effect of doxycycline on the cellular morphology of NCI-H446 and A549 by HCS. In response to doxycycline treatment, the perimeter-to-area ratio decreased, whereas pyknosis increased in both NCI-H446 and A549 cells (Fig. 3C & 3F). The relative area of nucleus also increased, whereas the DNA content in cells decreased in both NCI-H446 and A549 cells (Fig. 3D & 3E). As shown in Fig. 3, the results of DNA reduction were comparisons of all cells, not only S stage cells. For A549 cells, as shown in Fig. 1E, the number of S stage cells did not differ between groups.

### Doxycycline inhibits the activity of EMT transcription factors

**Figure 4:**
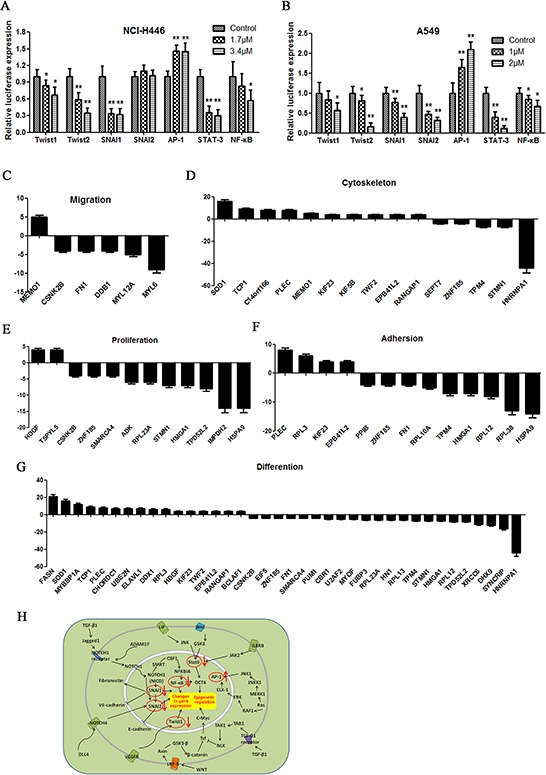
Dual luciferase assay results **A, B.** Dual-luciferase assay results for NCI-H446 and A549 cells suggesting that Twist1, Twist2, SNAI1, SNAI2, AP-1, STAT-3, and NF-κB are target genes of doxycycline. Doxycycline had different effects on transcript activation in different cells. In NCI-H446, doxycycline inhibited SNAI2. **C–G.** Multidimensional liquid chromatography-tandem mass spectrometry was performed to evaluate the differentially expressed proteins of NCI-H446 cells. Among the 78 differential proteins, 36 were upregulated and 42 were downregulated. These proteins are involved in migration, cytoskeleton, proliferation, adhesion and differentiation. **H.** Doxycycline signaling pathway. Similar results were obtained from three independent experiments. Each experiment was performed in triplicate. Results show the means of the three experiments, and the error bars represent standard deviation (*P < 0.05 and **P < 0.01).

NCI-H446 and A549 cells transfected with luciferase reporter plasmids for various gene promoters or response elements were treated with doxycycline for 48 h. In NCI-H446 cells, doxycycline suppressed the expression of Twist1, Twist2, SNAI1, STAT-3, and NF-κB and promoted the expression of AP-1. However, no change in SNAI2 expression was observed (Fig. 4A). In A549 cells, doxycycline suppressed the expression of Twist1, Twist2, SNAI1, SNAI2, STAT-3, and NF-κB and promoted the expression of AP-1 (Fig. 4B). Multidimensional liquid chromatography-tandem mass spectrometry was performed to evaluate differentially expressed proteins in NCI-H446 cells after doxycycline treatment. 36 proteins were upregulated by doxycycline and 42 were downregulated. After doxycycline treatment in cancer cell lines, RPLs were directly or indirectly regulated. Some RPLs were inhibited by doxycycline, such as RPL23A, RPL10A, RPL12, RPL38 and RPL13. On the contrary, RPL3 was compensatorily increased to maintain basic protein synthesis. (Fig. 4C–4G). These proteins are involved in cell migration, cytoskeletal maintenance, cell proliferation, adhesion, and differentiation (Fig. 4H).

### Doxycycline has an antitumor effect in a mouse xenograft model

**Figure 5:**
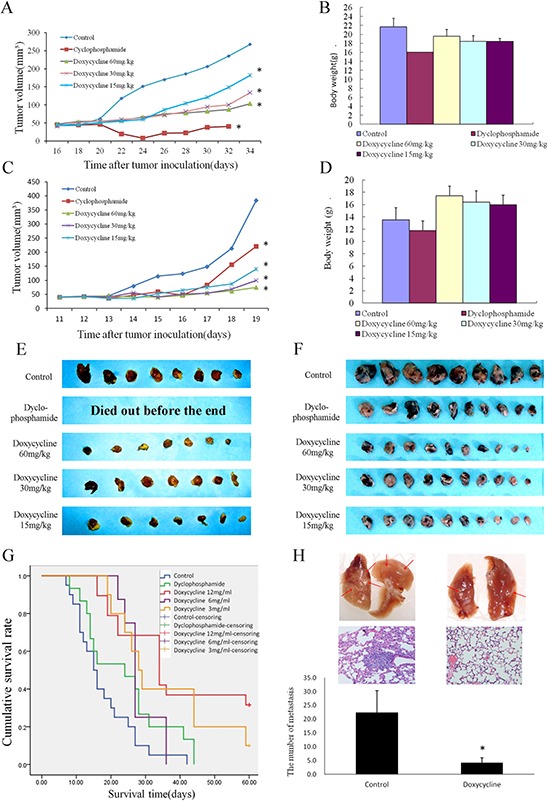
Effect of doxycycline on a nude mouse xenograft model. Mice were treated with saline, cyclophosphamide (20 mg/kg), or doxycycline (60, 30, or 15 mg/kg) for 5 weeks **A.** Changes in tumor volume of NCI-H446 xenografts. Both doxycycline and cyclophosphamide diminished tumor volume of the tumors. **B.** Body weights (g) of animals with NCI-H446 xenografts. Doxycycline increased the body weights of nude mice with xenografts compared with cyclophosphamide. **C.** Changes in tumor volume of Lewis lung carcinoma (LLC) xenografts. Both doxycycline and cyclophosphamide diminished tumor volume. **D.** Body weights (g) of animals with LLC xenografts. Doxycycline increased the body weights of nude mice of xenografts compared with cyclophosphamide. **E.** Doxycycline treatment inhibited NCI-H446 xenografts growth in a dose-dependent manner. Particularly, the cyclophosphamide xenografts died out before the end of the experiment because of toxicity. **F.** Doxycycline treatment inhibited LLC xenografts growth in a dose-dependent manner. **G.** The median survival time of control groups of LLC xenografts is 15.5 days. The median survival times of high-dose, middle-dose, and low-dose doxycycline groups were 235%, 197%, and 184%, respectively, compared with the control group. By contrast, the median survival time of the cyclophosphamide treatment was increased by 171%. **H.** The number of tumors that shifted stoves markedly decreased in lungs of nude mice with LLC xenografts. Each experiment was performed in triplicate. Results show the means of the three experiments, and the error bars represent standard deviation (*P < 0.05).

We next examined the effects of doxycycline on spontaneous lung metastasis using NCI-H446 human lung cancer and Lewis lung carcinoma (LLC) xenografts in nude mice. Doxycycline treatment inhibited tumor growth in a dose-dependent manner. In particular, the NCI-H446 xenografts in the cyclophosphamide group died out before the end of the experiment (Fig. 5A & 5C, 5E & 5F). Body weight increased in the doxycycline-treated group (Fig. 5B & 5D). The median survival time of the control groups of LLC xenografts was 15.5 days. When compared with the control group, the median survival times of high-dose, middle-dose, and low-dose doxycycline treatment groups were increased by 235%, 197%, and 184%, respectively, and the median survival time of the cyclophosphamide treatment group was increased by 171% (Fig. 5G). The number of tumors that shifted stoves was decreased from 22.4 ± 7.96 to 4.20 ± 1.79 in the lungs of nude mice with LLC xenografts treated with doxycycline, (Fig. 5H). These results strongly suggest that doxycycline inhibits tumor growth and metastasis.

### Doxycycline alters the expression of EMT markers in cancer tissues

**Figure 6:**
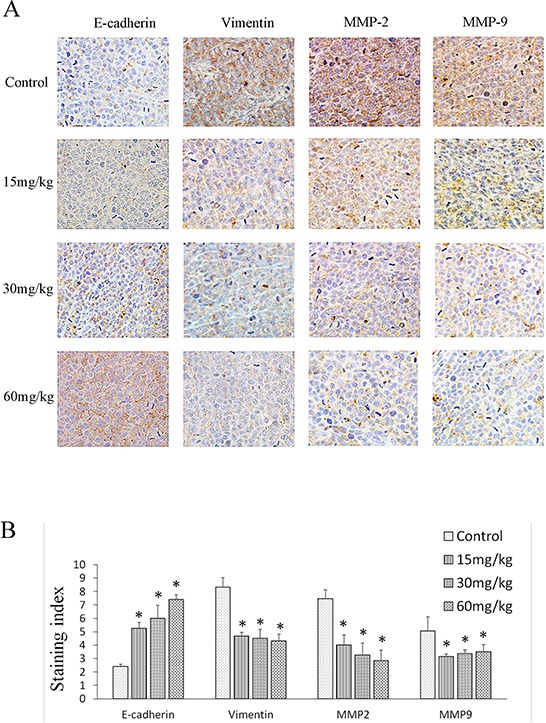
Effect of doxycycline on EMT protein expression. Brown or yellow staining was observed in the cytoplasm or nucleus E-cadherin was located in membranes, and vimentin, MMP-2, and MMP-9 were found in the cytoplasm. **A.** Representative photographs of treated and untreated cells. **B.** Doxycycline-treated sections had weaker vimentin, MMP-2, and MMP-9 staining but stronger E-cadherin staining when compared with the sections obtained from control mice. Each experiment was performed in triplicate. Results show the means of the three experiments, and the error bars represent standard deviation (*P < 0.05)

Immunohistochemical staining for E-cadherin, vimentin, MMP-2, and MMP-9 showed their expression to be correlated with doxycycline treatment. E-cadherin exhibited low expression, whereas vimentin, MMP-2, and MMP-9 exhibited high expression in tumor cells treated with doxycycline compared with the untreated group in both the membrane and cytoplasm (Fig. 6A & 6B).

## DISCUSSION

By preventing and suppressing tumor invasion and metastasis it may be possible to decrease the mortality rates of patients with malignant tumors [13]. Despite efforts to develop alternative treatments for lung cancer [14, 15], in many cases the cancers are refractory and there is still no effective therapy [16, 17, 18, 19].

Doxycycline has been shown to have high anti-metastatic activity and low cytotoxicity in melanoma and breast carcinoma [20, 21]. In clinical trials with patients with pleural effusion, satisfactory results were achieved when doxycycline was used and there was inhibition of the activity of the suspended tumor cells. The current study focuses on the mechanism and anti-metastatic effects of doxycycline. We used several human cancer cell lines to investigate the effect of doxycycline on cell viability and the cell cycle. Our results showed that doxycycline inhibited the proliferation of NCI-H446 and A549 cells more than the other cell lines. Doxycycline has a strong inhibitory effect on malignancies originating from mesenchyme and neuroectoderm, including melanomas, endothelial cell tumors, and sarcomas [20, 22, 23]. All three kinds of lung cancer cells used in this study have mesodermal characteristics and readily undergo EMT [24–26]. NCI-H446 is a small cell lung cancer cell originating from neuroectoderm, while A549 cells are thought to have already undergone EMT. By contrast, lung squamous cell carcinoma is not very sensitive to doxycycline (unpublished data).

Doxycycline has a strong inhibitory effect on cells with mesenchymal characters. Early studies showed that doxycycline influences focal adhesion kinase (FAK) and cell adhesion and causes cells to lose anchors during migration [20]. Our current study does not examine whether doxycycline directly inhibits chemotaxis or indirectly inhibits chemotaxis by the inhibition of cell adhesion. It is possible that increased invasion and migration might due to increased generation of chemokines from tumor cells. Doxycycline also has other targets, including MMPs and E-cadherin, ultimately inhibiting metabolism and metastasis [27]. Two members of the MMP family, MMP-2 and MMP-9, are necessary for tumor invasion and metastasis [28, 29] [30]. Therefore, decreasing MMP activity could inhibit cancer cell invasion and metastasis [31, 32]. We examined the regulation of MMP activity after doxycycline treatment. Our data show that doxycycline inhibits MMP-2 and MMP-9 activation, which is consistent with its inhibitory effects on metastasis.

EMT initiates tumor metastasis and alters cell-cell adhesion [33–35]. E-cadherin and vimentin, two biomarkers of EMT, mediate cell adhesion. Downregulation of E-cadherin expression is both necessary and sufficient to confer metastatic ability to lung cancer cells that are otherwise non-metastatic [36, 37]. Vimentin is an intermediate filament protein that, along with microtubules and actin filaments, forms the cytoskeleton. In some carcinomas, including prostatic carcinoma, vimentin is overexpressed and is therefore a marker for invasiveness and metastasis [38]. In this study, we found that doxycycline increased E-cadherin levels and decreased vimentin protein expression. This finding suggests that doxycycline suppresses EMT in lung cancer. In addition, studies of promoter activation and proteomics after doxycycline treatment revealed that doxycycline inhibits EMT-related transcription factor activity and that doxycycline exerts its antitumor effect by interfering with tumor cell EMT. These findings show that doxycycline acts upstream of EMT-related signal transduction to inhibit a wide range of cellular functions. The inhibitory effects of doxycycline on bacteria is derived from its interference with rhibosomes, which result in the inhibition of protein synthesis. After doxycycline treatment in cancer cell lines, RPLs were directly or indirectly regulated. Some RPLs were inhibited by doxycycline, such as RPL23A, RPL10A, RPL12, RPL38 and RPL13. On the contrary, RPL3 was compensatorily increased to maintain basic protein synthesis. As shown in Table S1, many molecular and biological functions of these RPLs are similar even the same between Eukaryotes and prokaryotes. In summary, doxycycline may be an effective alternative treatment for persistent carcinoma, as well as a new candidate for adjuvant chemoradiotherapy.

## MATERIALS AND METHODS

### Materials

Crystal violet, doxycycline hydrochloride, and 3-(4,5-dimethylthiazol-2-y1)-2,5-diphenyltetrazolium bromide (MTT) were purchased from Sangon Biotech (Shanghai, China). Matrigel and transwell chambers were purchased from BD Biosciences (San Jose, CA, USA). Propidium iodide (PI) was purchased from Beyotime Biotech (Jiangsu, China). The antibodies to MMP-2, MMP-9, vimentin, and E-cadherin were purchased from Affinity Bioreagents (Colorado, USA). Rabbit polyclonal anti-E-cadherin and rabbit polyclonal anti-MMP-9 were purchased from ZSGB-BIO (Beijing, China). Fluorescein isothiocyanate AffiniPure Goat Anti-Rabbit IgG (H+L) secondary antibodies were purchased from EarthOx (San Francisco, USA). Dual luminescence assay kit was purchased from GeneCopoeia (Guangzhou, China).

### Cell culture

The following human cell lines were obtained KeyGen Biotech (Nanjing, China): NCI-H446, and A549 lung cancer cell lines; PLC, SMMC7721, HepG2, MHCC97H, and MHCC97L hepatoma carcinoma lines; LOVO colon cancer line; PC-3 prostate cancer cell line; A875, A375, Mum2B, and Mum2C melanoma lines; MCF-7 and MDA-MB-231 breast cancer cell lines; SGC-7901gastric cancer cell line; PCNA-1 and AsPC-1 pancreatic cancer cell lines; HeLa cervical cancer line; K562 and HL60 leukemia lines; and SH-SY5Y neuroblastoma line. Cells were cultured in medium supplemented with 10% heat-inactivated (56°C, 30 min) fetal calf serum (Hyclone, USA) and maintained at 37°C with 5% CO_2_ in a humidified atmosphere. Details are shown in Table S2.

### Cell viability assay

Cell viability was determined by MTT assay. Cells (5 × 10^3^ cells/mL) were seeded in 96-well culture plates. After overnight incubation, cells were treated with various concentrations of doxycycline hydrochloride. After 48 h incubation, cell viability was measured after the addition of 20 μL MTT at 37°C for 4 h. Afterward, 150 μL dimethyl sulfoxide was added to dissolve the formazan crystals. Optical density was measured at 570 nm with a microplate reader (Multiskan™ FC, Thermo Scientific, Waltham, MA, USA).

### Wound-healing assay

NCI-H446 and A549 cells were grown on a 35 mm dish to 100% confluence and then scratched to form a 100-μm wound using sterile pipette tips. The cells were then cultured in the presence or absence of doxycycline hydrochloride in serum-free media for 24 h. Images of the cells were taken at 24 and 48 h using a light microscope (Nikon, Japan).

### Invasion assays

Cell invasion assays were performed using a transwell chamber inserted with a polyethylene terephthalate filter membrane containing 8.0 μm pores in 24-well plates (Corning, USA). For cell invasion assays, the filter membranes were coated with Matrigel. Cells (1 × 10^5^ cells/mL) suspended in 200 μL of serum-free medium were seeded onto the upper compartment of the transwell chamber. The lower chamber was filled with medium containing 10% fetal bovine serum (as chemoattractant for migrated and invaded cancer cells) and various concentrations of doxycycline hydrochloride. After incubation for 24 h, the medium in the upper chamber was removed, and the filters were fixed with 10% methanol for 20 min. The cells remaining on the upper surface of the filter membrane were then completely removed by wiping with a cotton swab, and the cells on the opposite surface of the filter membrane were stained with 0.1% crystal violet for 10 min. The invading cells were then visualized and counted from six randomly selected fields using an inverted microscope at 100 × magnification.

### Fluorescence-activated cell sorting (FACS) analysis

NCI-H446 and A549 cells were harvested separately and washed twice with phosphate buffered saline (PBS). The cells were then fixed with cold 70% ethanol for 12 h at 4°C. Afterward, the cells were washed with 1 mL PBS. The cells were treated with 500 μL PBS containing 100 μg/mL ribonuclease and 50 μg/mL PI. The cells were analyzed by flow cytometry (Millipore guava easyCyte™).

### Immunofluorescent staining

NCI-H446 and A549 cells (4 × 10^3^ cells/mL) were seeded in 96-well culture plates. After overnight incubation, the cells were treated with various concentrations of doxycycline hydrochloride. After incubation for 24 h, cells were washed twice in PBS, fixed with 10% formalin in PBS, permeabilized, and blocked with PBS containing NP-40 (0.1%) and bovine serum albumin (3%). The cells were then incubated in the same solution containing primary antibodies specific for either E-cadherin antibody (1:50 dilution) or vimentin antibody (1:50 dilution) for 1 h at room temperature (25°C). Cells were washed four times in PBS and incubated in secondary antibody (1:200 dilution) for 30 min at room temperature. Cells were then washed four times in PBS and covered with Hoechst 33342 dye for 30 min at room temperature. Cells were washed four additional times in PBS then proteins were visualized with high-content screening (HCS) systems.

### Gelatin zymography assay

NCI-H446 and A549 cells (1 × 10^6^ cells/well) were plated in 12-well plates and incubated in serum-free RPMI 1640 medium in the presence of doxycycline hydrochloride for 24 h. At the end of incubation, the conditioned medium was harvested, placed on 10% sodium dodecyl sulfate (SDS)-polyacrylamide gel containing 0.2% gelatin (Sigma-Aldrich Corp), and then separated by electrophoresis. The gels were soaked in 2.5% Triton X-100 in deionized water twice for 60 min at 25°C to remove SDS. Gels were incubated at 37°C with substrate buffer (50 mM Tris HCl, 5 mM CaCl_2_, 0.02% NaN_3_, and 1% Triton X-100, pH 8.0) for 18 h. The gel was stained using 0.2% Coomassie blue for 1 h and destained in water containing 10% acetic acid and 50% methanol. Bands corresponding to the activity of MMP-2 and MMP-9 were quantified with ImageJ (National Institutes of Health).

### Dual-luciferase assay

NCI-H446 and A549 cells were transfected with dual-reporter constructs using transfection reagents. After changing to fresh medium 24 h after transfection, cells were treated with various concentrations of doxycycline hydrochloride. After 48 h, the culture medium was collected into a 96-well white plate and luminescence measured with a luminometer. Details are shown in Table S3 and S4.

### Multidimensional liquid chromatography-tandem mass spectrometry

NCI-H446 and A549 cells (4 × 10^3^ cells/mL) were seeded in a 100 mm dish to 70–80% confluence. The cells were then cultured in the presence (3.4 μM) or absence of doxycycline hydrochloride for 24 h. After cell lysis, samples were tested with multidimensional liquid chromatography-tandem mass spectrometry.

### Animal studies

Male BAlB/c nu/nu mice, 5–6 weeks old, were maintained in a specific pathogen-free animal care facility according to institutional guidelines. Xenografts of tumors were established by subcutaneous injection of 1 × 10^7^ cells (suspended in PBS) into the flank. One day after tumor cell inoculation, the mice were randomly divided into 5 groups (*n* = 10/group). After the tumors reached an approximate volume of 100 mm^3^ (approximately six weeks after injection), the mice were treated with 60, 30, or 15 mg/kg of doxycycline; 20 mg/kg of cyclophosphamide; or saline by oral gavage once a day. Body weights were measured at different time points after tumor cell inoculation. Tumor diameters were measured every day, and tumor volumes were calculated according to the formula *V* = ab^2^/2 (*a* = length of tumor, *b* = width of tumor). Seven weeks after treatment, all mice were euthanized and both xenografts and lungs were resected and measured. Lung tissue was harvested for histologic examination and the nodes in lungs were observed using a stereoscopic microscope. Metastases from xenograft to lungs were measured after HE staining.

Another 50 mice were allocated randomly to 5 groups as described above (10 mice per group), in order to measure survival rates. Each mouse was injected 1 × 10^7^ cells (suspended in PBS) in the caudal vein. The survival time of every mouse were recorded.

### Immunohistochemical analysis

Fresh tissues from mice were fixed in 4% paraformaldehyde, embedded in paraffin, cut into 4 μm thick slices, and placed on slides. The tissues were deparaffinized with xylene, dehydrated in decreasing concentrations of ethanol, and subsequently incubated with 3% hydrogen peroxide for 15 min to block endogenous peroxidase activity. For antigen retrieval, tissues were treated with citrate buffered saline (pH 6.0) for 15 min at 95°C. Tissues were incubated with normal goat serum for 20 min at room temperature to block unspecific labeling and then incubated with the following primary antibodies in a humidified chamber overnight at 4°C: rabbit polyclonal anti-E-cadherin (Zhongshan, ready-to-use), goat polyclonal anti-vimentin (Affinity, dilution 1:50), and rabbit polyclonal anti-MMP-9 (Zhongshan, ready-to-use). Diaminobenzidine and hematoxylin were used for color development and as counterstain, respectively. Expression of E-cadherin and vimentin were independently evaluated by two investigators. Tumor cells with brown staining of the cytoplasm, nucleus or membrane were considered positive and then scored based on four classes: none (0), weak brown (1+), moderate brown (2+), and strong brown (3+). The percentage of stained tumor cells was divided into five classes: 0 for negative cells, 1 for 1–25%, 2 for 25–50%, 3 for 50–75%, and 4 for > 75%.

### Statistical analyses

All data are expressed as means ± standard deviation. Comparisons between groups were performed by one-way analysis of variance followed by Bonferroni post hoc test (SPSS software package version 17.0, SPSS Inc., Chicago, IL, USA). The level of significance was set at *P* < 0.05.

## SUPPLEMENTARY FIGURE AND TABLES


