# Supplementary material for: Doxycycline reverses epithelial-to-mesenchymal transition and suppresses the proliferation and metastasis of lung cancer cells
Source: Oncotarget. 2015 Oct 14;6(38):40667–79. doi: 10.18632/oncotarget.5842 (PMC4747360; doi:10.18632/oncotarget.5842)
Supplement: Supplementary file 1 [file oncotarget-06-40667-s001.pdf]

SUPPLEMENTARY FIGURE AND TABLES

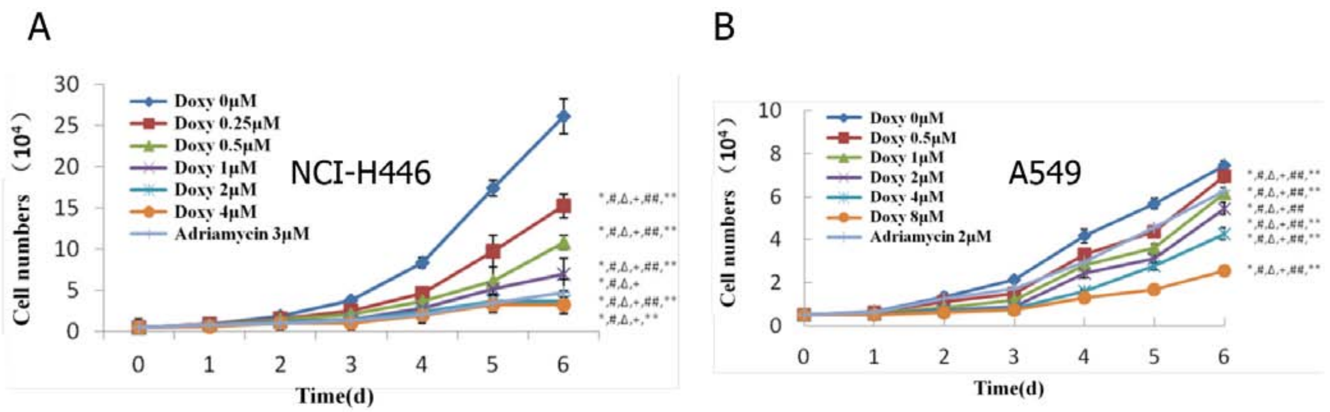

Supplementary Figure S1: The cell growth curves of NCI-H446 and A549 treated with indicated concentration of doxycycline and adriamycin. A. The doubling time of NCI-H446 in the first group (Doxy 0 μM) was 13.81 h. **B.** The doubling time of A549 in the first group (Doxy 0 μM) was 41.17 h.

Supplementary Table S1: Molecular and biological function of RPLs

RPLs	Molecular and biological function
RPL10A	poly(A) RNA binding, anatomical structure morphogenesis, cellular protein metabolic process, gene expression [1, 2]
RPL12	poly(A) RNA binding, cellular protein metabolic process [3]
RPL13	poly(A) RNA binding, RNA binding, cellular protein metabolic process [1, 4]
RPL23A	nucleotide binding, poly(A) RNA binding, rRNA binding, cell proliferation, cellular protein metabolic process [1, 3, 5]
RPL38	RNA binding, 90S pre-ribosome assembly, axial mesoderm development [6]
RPL3	poly(A) RNA binding, RNA binding, cellular protein metabolic process [1, 7]

1. Castello, A., et al., *Insights into RNA biology from an atlas of mammalian mRNA-binding proteins*. Cell, 2012. 149(6): p. 1393–406.

2. Fisicaro, N., et al., *Identification of genes downregulated in the thymus by cyclosporin-A: preliminary characterization of clone CSA-19*. Mol Immunol, 1995. 32: p. 565–72.

3. Baltz, A.G., et al., *The mRNA-bound proteome and its global occupancy profile on protein-coding transcripts*. Mol Cell, 2012. 46: p. 674–90.

4. Kenmochi, N., et al., *A map of 75 human ribosomal protein genes*. Genome Res, 1998. 8(5): p. 509–23.

5. Jiang, H., et al., *Suppression of human ribosomal protein L23A expression during cell growth inhibition by interferon-beta*. Oncogene, 1997. 14: p. 473–80.

6. Espinosa, L., et al., *Primary sequence of the human, lysine-rich, ribosomal protein RPL38 and detection of an unusual RPL38 processed pseudogene in the promoter region of the type-1 angiotensin II receptor gene*. Biochim Biophys Acta, 1997. 1354: p. 58–64.

7. Ou, J.H., et al., *Cloning and characterization of a human ribosomal protein gene with enhanced expression in fetal and neoplastic cells*. Nucleic Acids Res, 1987. 15(21): p. 8919–34.

Supplementary Table S2: Overview of the cell lines

Cell lines	Source	Growth Properties	Morphology	Propagation			Subculturing	Preservation	
				Medium	T	Atmosphere		Medium	T
NCI-H446	lung cancer	adherent	epithelial	RPMI1640+10%FBS	37°C	95% air<br>5% CO_2	remove medium<br>rinse with 0.25% trypsin, 0.03% EDTA solution<br>remove the solution and add an additional 1 to 2 ml of trypsin-EDTA solution<br>allow the flask to sit at room temperature until the cells detach<br>add fresh culture medium, aspirate and dispense into new culture flasks<br>Subcultivation ratio: 1:3 to 1:5<br>Medium renewal: 2 to 3 times per week	Complete growth medium supplemented with 10% DMSO	liquid nitrogen vapor phase
A549	lung cancer	adherent	epithelial	RPMI1640+10%FBS					
PLC	liver cancer	adherent	epithelial	RPMI1640+10%FBS					
SMMC-7721	liver cancer	adherent	epithelial	RPMI1640+10%FBS					
HepG-2	liver cancer	adherent	epithelial	DMEM (high glucose)+10%FBS+1% non-essential amino					
MHCC97H	liver cancer	adherent	epithelial	DMEM (high glucose)+10%FBS					
MHCC97L	liver cancer	adherent	epithelial	DMEM (high glucose)+10%FBS					
LOVO	colon cancer	adherent	epithelial	DMEM (high glucose)+10%FBS					

(*Continued*)

Cell lines	Source	Growth Properties	Morphology	Propagation			Subculturing	Preservation	
				Medium	T	Atmosphere		Medium	T
PC-3	prostate cancer	adherent	epithelial	F12K+10%FBS					
A875	melanoma	adherent	epithelial	DMEM (high glucose)+10%FBS					
A375	melanoma	adherent	epithelial	DMEM (high glucose)+10%FBS					
Mum2B	melanoma	adherent	epithelial	RPMI1640+10%FBS					
Mum2C	melanoma	adherent	epithelial	RPMI1640+10%FBS					
**Cell lines**	**Source**	**Growth Properties**	**Morphology**	**Propagation**			**Subculturing**	**Preservation**	
				**Medium**	**T**	**Atmosphere**		**Medium**	**T**
MCF-7	breast cancer	adherent	epithelial	RPMI1640+10%FBS+0.01 mg/ml bovine insulin	37°C	95% air5% CO_2	remove medium<br>rinse with 0.25% trypsin, 0.03% EDTA solution<br>remove the solution and add an additional 1 to 2 ml of trypsin-EDTA solution<br>allow the flask to sit at room temperature until the cells detach<br>add fresh culture medium, aspirate and dispense into new culture flasks<br>Subcultivation ratio: 1:3 to 1:5<br>Medium renewal: 2 to 3 times per week	Complete growth medium supplemented with 10% DMSO	liquid nitrogen vapor phase

(*Continued*)

Cell lines	Source	Growth Properties	Morphology	Propagation			Subculturing	Preservation	
				Medium	T	Atmosphere		Medium	T
MDA-MB-231	breast cancer	adherent	epithelial	DMEM (high glucose)+10%FBS					
SGC-7901	stomach cancer	adherent	epithelial	RPMI1640+10%FBS					
PANC-1	pancreatic cancer	adherent	epithelial	DMEM (high glucose)+10%FBS					
ASPC-1	pancreatic cancer	adherent	epithelial	DMEM (high glucose)+10%FBS					
HeLa	cervical cancer	adherent	epithelial	DMEM (high glucose)+10%FBS					
SH-SY5Y	Neurobla-stoma	adherent and suspension	epithelial	RPMI1640+10%FBS					
K562	leukemia	suspension	lymphoblast	RPMI1640+10%FBS			Start new cultures at 1 * 10^5 viable cells/ml. Subculture at 1 * 10^6 cells/ml. Medium renewal: Every 2 to 3 days		
HL60	leukemia	suspension	myeloblastic	RPMI1640+10%FBS					

Supplementary Table S3: Twist1, Twist2, SNAI1, SNAI2 promoter reporter clones

Promoter reporter clones	Promoter sequence	Vector information
Twist1*	Promoter Length: 1282 bp	pEZX-PG04
	Sequence length upstream of TSS: 1043 bp	
	Sequence length downstream of TSS: 238 bp	
Twist2*	Promoter Length: 1512 bp	
	Sequence length upstream of TSS: 1416 bp	
	Sequence length downstream of TSS: 95 bp	
SNAI1*	Promoter Length: 1255 bp	
	Sequence length upstream of TSS: 1185 bp	
	Sequence length downstream of TSS: 69 bp	
SNAI2*	Promoter Length: 1399 bp	
	Sequence length upstream of TSS: 1235 bp	
	Sequence length downstream of TSS: 163 bp	

*The promoter reporter clones were purchased from GeneCopoeia (Guangzhou, China)

Supplementary Table S4: AP-1, STAT3, NF-κB luciferase reporter gene vector

Reporter gene vector	Response element	Vector information
pAP1-TA-luc*	AP1 response element 26–67<br>TGACTAATGACTAATGACTAATGACTAATGACTAATGACTAA	pGL6-TA
pSTAT3-TA-luc*	STAT3 response element 32–86<br>TGCTTCCCG AACGTTGCTT CCCGAACGTT GCTTCCCGAA CGTTGCTTCC GAACGT	pGL6-TA
pNFκB-TA-luc*	NFκB response element 26–65<br>GGGAATTTCCGGGAATTTCCGGGAATTTCCGGGAATTTCC	pGL6-TA
pRL-TK[#]	HSV-TK promoter 7–759	pRL

[*]The reporter gene vector were purchased from Beyotime Biotechnology (Shanghai, China)
[#]The reporter gene vector was purchased from Promega
